# Treatment-associated polymorphisms in protease are significantly associated with higher viral load and lower CD4 count in newly diagnosed drug-naive HIV-1 infected patients

**DOI:** 10.1186/1742-4690-9-81

**Published:** 2012-10-03

**Authors:** Kristof Theys, Koen Deforche, Jurgen Vercauteren, Pieter Libin, David AMC van de Vijver, Jan Albert, Birgitta Åsjö, Claudia Balotta, Marie Bruckova, Ricardo J Camacho, Bonaventura Clotet, Suzie Coughlan, Zehava Grossman, Osamah Hamouda, Andrzei Horban, Klaus Korn, Leondios G Kostrikis, Claudia Kücherer, Claus Nielsen, Dimitrios Paraskevis, Mario Poljak, Elisabeth Puchhammer-Stockl, Chiara Riva, Lidia Ruiz, Kirsi Liitsola, Jean-Claude Schmit, Rob Schuurman, Anders Sönnerborg, Danica Stanekova, Maja Stanojevic, Daniel Struck, Kristel Van Laethem, Annemarie MJ Wensing, Charles AB Boucher, Anne-Mieke Vandamme

**Affiliations:** 1 Rega Institute for Medical Research, Katholieke Universiteit Leuven, Leuven, Belgium; 2 MyBioData, Rotselaar, Belgium; 3 Department of Virology, Erasmus Medical Center, Rotterdam, the Netherlands; 4 Clinical Microbiology, Karolinska University Hospital and Department of Microbiology, Tumor and Cell Biology, Karolinska Institute, Stockholm, Sweden; 5 Section for Microbiology and Immunology, Gade institute, University of Bergen, Bergen, Norway; 6 University of Milan, Milan, Italy; 7 National Institute of Public Health, Prague, Czech Republic; 8 Centro de Malária e outras Doenças Tropicais, Instituto de Higiene e Medicina Tropical, Universidade Nova de Lisboa, Lisbon, Portugal; 9 Centro Hospitalar de Lisboa Ocidental, Lisbon, Portugal; 10 irsiCaixa AIDS Research Institute & Lluita contra la SIDA Foundation, Hospital Universitari “Germans Trias i Pujol”, Badalona, Spain; 11 University College Dublin, Dublin, Ireland; 12 Sheba Medical Center, Tel-Hashomer, and School of Public Health, Tel-Aviv University, Tel-Aviv, Israel; 13 Robert-Koch Institute, Berlin, Germany; 14 Warsaw Medical University and Hospital for Infectious Diseases, Warsaw, Poland; 15 Institut für Klinische und Molekulare Virologie, University of Erlangen, Erlangen, Germany; 16 University of Cyprus, Nicosia, Cyprus; 17 Statens Serum Institute, Copenhagen, Denmark; 18 National Retrovirus Reference Center, Department of Hygiene Epidemiology of Medical Statistics, University of Athens, Medical School, Athens, Greece; 19 University of Ljubljana, Ljubljana, Slovenia; 20 Medical University of Vienna, Vienna, Austria; 21 National Institute of Health and Welfare, Helsinki, Finland; 22 Centre Hospitalier de Luxembourg and Centre de Recherche Public de la Santé, Luxembourg, Luxembourg; 23 Department of Medical Microbiology, University Medical Center Utrecht, Utrecht, the Netherland; 24 Divisions of Infectious Diseases and Clinical Virology, Karolinska Institutet, Stockholm, Sweden; 25 Slovak Medical University, Bratislava, Slovak Republic; 26 School of Medicine, University of Belgrade, Belgrade, Serbia

## Abstract

**Background:**

The effect of drug resistance transmission on disease progression in the newly infected patient is not well understood. Major drug resistance mutations severely impair viral fitness in a drug free environment, and therefore are expected to revert quickly. Compensatory mutations, often already polymorphic in wild-type viruses, do not tend to revert after transmission. While compensatory mutations increase fitness during treatment, their presence may also modulate viral fitness and virulence in absence of therapy and major resistance mutations. We previously designed a modeling technique that quantifies genotypic footprints of in vivo treatment selective pressure, including both drug resistance mutations and polymorphic compensatory mutations, through the quantitative description of a fitness landscape from virus genetic sequences.

**Results:**

Genotypic correlates of viral load and CD4 cell count were evaluated in subtype B sequences from recently diagnosed treatment-naive patients enrolled in the SPREAD programme. The association of surveillance drug resistance mutations, reported compensatory mutations and fitness estimated from drug selective pressure fitness landscapes with baseline viral load and CD4 cell count was evaluated using regression techniques. Protease genotypic variability estimated to increase fitness during treatment was associated with higher viral load and lower CD4 cell counts also in treatment-naive patients, which could primarily be attributed to well-known compensatory mutations at highly polymorphic positions. By contrast, treatment-related mutations in reverse transcriptase could not explain viral load or CD4 cell count variability.

**Conclusions:**

These results suggest that polymorphic compensatory mutations in protease, reported to be selected during treatment, may improve the replicative capacity of HIV-1 even in absence of drug selective pressure or major resistance mutations. The presence of this polymorphic variation may either reflect a history of drug selective pressure, i.e. transmission from a treated patient, or merely be a result of diversity in wild-type virus. Our findings suggest that transmitted drug resistance has the potential to contribute to faster disease progression in the newly infected host and to shape the HIV-1 epidemic at a population level.

## Background

Following initial HIV-1 infection, the rate of clinical disease progression reflects the complex interplay of host- and virus-related as well as socio-economic factors. This highly variable rate can be assessed and predicted by monitoring the evolution of prognostic markers such as the number of viral particles in the plasma (viral load or viremia) and CD4+ T-lymphocytes cell count (CD4 count). Constituting the only current strategy to delay disease progression, the primary goal of antiretroviral therapy (ART) is to maximally inhibit viral replication and to aim for immunological reconstitution. However, accumulation of drug resistance mutations during suboptimal therapy severely affects the clinical benefit of ART, leading to therapy failure [1].

HIV-1 evolutionary dynamics under selective pressure of ART are largely governed by competitive fitness, to which viral replication, phenotypic drug resistance and intrinsic replicative capacity (RC) contribute. While an increased ability of the virus to replicate in the presence of drug results from decreased phenotypic drug susceptibility, major drug resistance mutations reduce the inherent ability of HIV-1 to replicate in absence of drug (replicative capacity). Hence, virus evolution is characterized by repair strategies that include compensatory mutations in the targeted gene [2]. Despite these compensatory effects, drug-resistant viruses tend to replicate less efficiently than wild-type viruses in absence of treatment, which is exemplified by the fact that archived wild-type viruses become again predominant during treatment interruption [3].

The transmission of drug resistance (TDR) among adults recently infected in North-America and Europe is a consequence of the widespread use of antiviral agents and related resistance accumulation in the ART-experienced population. A large survey of 17 European countries reported a TDR prevalence of 9.0% among newly diagnosed persons [4-6]. Transmitted resistant virus was initially believed to become irrelevant over time, as it would gradually disappear from the dominant quasispecies: mutations reverting to wild-type or alternative amino acids reflect the impaired fitness of TDR variants in absence of drug pressure and wild-type virus. However, persistence of TDR variants as dominant quasispecies, within a new host and within transmission chains, has been reported. Prevention of wild-type state reversion may result from absence of competition in the founder virus population [7], the existence of steep fitness valleys between resistance mutant and original wild-type [8], restoration of fitness through selection of compensatory mutations [9] or a combination of these factors. However, the relationship between fitness cost and persistence may be complex due to mutational interactions [10], and early immune responses may as well influence the reversion of TDR.

CD4 cell loss is a prognostic marker for development of clinical symptoms and progression towards AIDS. HIV-1 isolates vary widely in features that determine viral fitness and virulence. For HIV-1, it might seem reasonable to infer that increased viral fitness coincides with elevated pathogenicity, since an inverse relationship between the viral load and the rate of CD4 decline is often observed. Under such assumption, presence of transmitted drug resistance mutations that impair replication capacity could result in lower viral loads, thereby sustaining CD4 cell counts and delaying disease progression [11]. However, due to their high fitness costs in a drug free environment, major resistance mutations tend to revert after transmission. Although currently unknown, mutations selected during treatment for their compensatory fitness effects could be more persistent, since these mutations can also occur as natural polymorphisms [12]. Consequently, these compensatory mutations may modulate replication capacity of the virus not only in presence but also in absence of detrimental, major resistance mutations [13,14], and if so, it remains unclear whether their presence will result in higher viral fitness and virulence.

It has been observed that the viral genotype has a strong and direct effect on HIV-1 viral load [15]: the viral load of the transmitting partner is strongly predictive of the viral load in the recipient partner [16] and polymorphic variation associated with replicative capacity has been reported [17]. To what extent genetic variation resulting from transmitted drug resistance is influencing viral load or CD4 count in the recipient is not fully understood. Although transmission of drug resistance has been widely documented, whether drug selective pressure could shape the epidemic at a population level or alter the natural history of infection has been poorly investigated. In this study, we aimed to elucidate the potential of transmitted drug resistance to influence the natural history of HIV-1 infection at a population level. We investigated whether polymorphic compensatory mutations, reported to be enriched during therapy and therefore contributing to fitness under drug selective pressure, were also associated with increased fitness and virulence in patients never exposed to ART. For this purpose, we used a model that correlated fitness of genetic variants with drug selective pressure, and verified whether increased fitness under such a model also correlated with increased fitness in newly diagnosed treatment naive individuals, as measured by viral load and CD4 cell count. The effect of known TDR and compensatory mutations was also investigated in support of our hypothesis.

## Results

### Descriptive characteristics of study population

The analysis was restricted to HIV-1 subtype B sequences in order to minimize inter-subtype variability in the number and prevalence of polymorphic mutations and to exclude possible confounding effects on disease progression [18]. The analyzed SPREAD dataset contained 1782 newly diagnosed individuals that were infected with a HIV-1 subtype B virus (65%). A measurement of viral load and CD4 count was available for 1599 patients. Baseline characteristics of these patients are described in Table 1. Genotypic evidence of TDR was detected in 9.4% ( 95%-CI: 8.1-11.0) of patients, including 51 patients with PI (3.2%, 95%-CI: 2.4-4.2), 84 patients with NRTI (5.3%, 95%-CI: 4.2-6.4) and 41 patients with NNRTI (2.6%, 95%-CI: 1.9-3.5) resistance. Studies have suggested that patients with evidence of TDR may harbour virus with impaired replication capacity potentially leading to a less pathogenic virus. However, patients with or without indications of TDR had overall similar characteristics, and no significant difference in viral load (p-value = 0.52) and CD4 cell count (p-value = 0.14) could be detected.

**Table 1:** Characteristics of HIV-1 subtype B patients included in the analyses for prediction of viral load and CD4 cell count

**Characterististics**	**Subtype B patients (N = 1599)**	**TDR (N = 151)**	**Wild-type (N = 1446)**
log_10_ HIV-RNA copies/ml	4.85 (4.32 – 5.35)	4.83 (4.30 – 5.33)	4.86 (4.32 – 5.35)
CD4 cells/mm^3^	382 (212 – 583)	410 (249 – 582)	377 (209 – 583)
Age, years	35 (29 – 42)	34 (28 – 40)	35 (30 – 42)
Male sex , n (%)	1413 (89%)	137 (91%)	1276 (89%)
Duration of infection, n (%)			
< 1 year	541 (34%)	57 (37%)	484 (33%)
Undefined	1056 (66%)	94 (63%)	962 (67%)
Source of HIV-1exposure, n (%)			
Homo/bisexual contact	1016 (65%)	104 (69%)	912 (63%)
Heterosexual contact	123 (20%)	26 (17%)	290 (20%)
Intravenous drug use	316 (8%)	8 (5%)	115 (8%)
Other	142 (9%)	13 (9%)	129 (9%)
Area of origin, n (%)			
Western Europe	1147 (72%)	105 (70%)	1042 (72%)
Eastern Europe & Central Asia	282 (18%)	27 (19%)	255 (18%)
Other	168 (10%)	19 (11%)	149 (10%)

Patient characteristics are shown for the subtype B study dataset (N = 1599), including patients with genotypic evidence of transmitted drug resistance (TDR) and patients without genotypic evidence of transmitted drug resistance (wild-type). Data are expressed as median values accompanied by interquartile ranges, or as number of patients accompanied by proportion of the subtype B dataset (%).

### Estimating the fitness of the subtype B sequences under drug selective pressure

Since variation in viral load and CD4 cell count could not be explained by the presence or absence of TDR, we assessed to what extent variability in these baseline parameters could be explained by mutations and polymorphisms that contribute to increased in vivo fitness under drug selective pressure. To this aim, two fitness models (F_*PI*_ and F_*RTI*_) assigned fitness values to genetic variants. These models were estimated by relating the increase in prevalence of mutations with selective advantage during treatment (Additional file 1, [19]). Given that epistatic interactions alter the fitness impact of a mutation depending on the context of other mutations, we considered not only individual mutations, but also the increase in prevalence of mutation patterns. Consequently, the relative contribution of a mutation to the estimated fitness varied depending on to the presence of other mutations with which it interacts. The highly complex fitness function returned for each genotype a single fitness value based on the different mutations present in the genotype. Using the fitness models (F_*PI*_ and F_*RTI*_), we computed for each sequence the estimated in vivo fitness of respectively subtype B protease and reverse transcriptase. Mutations used in these models outnumbered the TDR mutation list [20], and included in addition to major resistance mutations also polymorphic, compensatory mutations (see Additional file 2 for a complete list of the fitness mutations and their prevalence in the study population). Both types of mutations were assigned higher weights in our fitness landscapes. Since it has been reported that major resistance mutations decrease replicative capacity in absence of selective pressure [1], we hypothesized that treatment-related polymorphisms (so-called compensatory mutations) may increase fitness also in absence of drug selective pressure. In one approach to evaluate the contribution of these compensatory mutations, a second fitness estimate was calculated for those sequences with major drug resistance mutations (11.4%) by reverting the major resistance mutation to the wild-type amino acid (F_*PI*−*m*_ and F_*RTI*−*m*_), thereby excluding the fitness contributions of major resistance mutations. The different fitness estimates of the sequences are summarized in Table 2, with the range of estimated fitness being more dispersed for PR than for RT. Computed fitness values were significantly higher in patients with TDR compared to patients without TDR, except for the estimated fitness of PR when the effect of major resistance mutations was excluded (log_10_F_*PI*−*m*_, p-value = 0.427).

**Table 2:** Estimated fitness according to the fitness landscape models

**Factors**	**Subtype B patients (N = 1599)**	**TDR (N = 151)**	**Wild-type (N = 1446)**	**p-value**
log_10_F_*PI*_	0.38 (0.24 – 0.56)	0.44 (0.26 – 0.66)	0.38 (0.24 – 0.56)	0.001
log_10_F_*PI*−*m*_	0.38 (0.24 – 0.55)	0.41 (0.25 – 0.57)	0.38 (0.24 – 0.56)	0.427
log_10_F_*RTI*_	0.04 (-0.03 – 0.11)	0.14 (0.03 – 0.27)	0.03 (-0.03 – 0.10)	< 0.001
log_10_F_*RTI*−*m*_	0.04 (-0.03 – 0.11)	0.08 (0.01 – 0.17)	0.03 (-0.03 – 0.11)	< 0.001

Log_10_F_*PI*_is the estimated PR fitness and log_10_F_*PI*−*m*_is the estimated PR fitness when the influence of major resistance mutations on fitness is excluded. Same nomenclature applies to fitness estimates of RT. Data are expressed as median values with the interquartile range between brackets.

### Evaluating genotypic predictors of viral load and CD4 cell count

The following genotypic predictors of viral load and CD4 were investigated: the presence of TDR, estimated fitness under a drug selective pressure model (log_10_F_*PI*_, log_10_F_*RTI*_) and the number of known compensatory mutations [21]. However, previous studies showed that levels of viral replication (i.e. viral load) and immune depletion (i.e. CD4 cell count decline) result from a complex interplay of between host and virus characteristics including time since infection, trends in TDR over calendar year, socio-economic factors, access to medical care and more. In our study, indications of acute infection at the time of diagnosis were significantly associated with higher viral loads (4.95 log_10_ copies/mL [IQR: 4.4-5.4 log_10_ copies/ml] versus 4.82 log_10_ copies/mL [IQR: 4.3-5.3 log_10_ copies/ml], p-value = 0.015) and higher CD4 counts (509 cells/mL [IQR: 360-661 cells/mL] versus 314 cells/mL [IQR: 134-503 cells/mL], p-value < 0.001). The presence of TDR was however not significantly higher in individuals recently infected than in individuals with unknown duration of infection, neither overall TDR (10.7% vs 8.9% respectively, p-value = 0.25), nor PI (4.2% versus 2.6%, p-value = 0.11) or RTI (7.0% vs 6.9%, p-value = 0.96) separately. The log fitness estimates and their range were also not significantly different (data not shown).

To account for duration of infection and other potential confounders, square-root transformed CD4 counts and log10 transformed viral load were modeled as a linear function of TDR or estimated in vivo fitness. Since especially recent infection was anticipated to bias the results, the simplest model (Model 1) included predictors derived from the genotype (TDR_*PI*_, TDR_*RTI*_, log_10_F_*PI*_, log_10_F_*RTI*_) and the duration of infection (recently infected vs unknown duration of infection). In the fully adjusted analyses (Model 2), risk group, age, country of origin and gender were added as explanatory variables (Table 3). In both models, there was no significant association between infection with TDR and viral load or CD4 count. However, higher estimated fitness under the PI but not under the RTI drug selective pressure model (log_10_F_*PI*_ respectively log_10_F_*RTI*_) was significantly correlated with higher viral load and lower CD4 count. To verify whether a single country introduced a bias, regression analyses were repeated by iteratively excluding data from one country, which did not change our results (data not shown). These observed correlations indicate that in treatment-naive patients, mutations in PR that give a selective advantage under drug selective pressure and are not included in the TDR list, are associated with better replicating virus in vivo.

**Table 3:** Regression analysis to predict viral load and CD4 count

	**Model 1**		**Model 2**	
**Factors**	**log VL (95% CI)**	**p-value**	**log VL (95% CI)**	**p-value**
TDR_*PI*_	-0.073 (-0.287 – 0.140)	0.500	-0.080 (-0.290 – 0.129)	0.451
TDR_*RTI*_	-0.009 (-0.157 – 0.138)	0.903	-0.003 (-0.147 – 0.142)	0.971
log_10_F_*PI*_	0.271 (0.122 – 0.420)	0.000	0.251 (0.104 – 0.398)	0.001
log_10_F_*RTI*_	-0.090 (-0.324 – 0.144)	0.449	-0.133 (-0.363 – 0.097)	0.258
log_10_F_*PI*−*m*_	0.314 (0.156 – 0.472)	0.000	0.294 (0.137 – 0.450)	0.000
log_10_F_*RTI*−*m*_	-0.117 (-0.459 – 0.223)	0.498	-0.180 (-0.516 – 0.155)	0.292
**Factors**	**sqrt CD4 (95% CI)**	**p-value**	**sqrt CD4 (95% CI)**	**p-value**
TDR_*PI*_	-0.357 (-2.313 – 1.600)	0.721	-0.501 (-2.417 – 1.416)	0.609
TDR_*RTI*_	0.507 ( -0.844 – 1.859)	0.462	0.362 (-0.963 – 1.688)	0.592
log_10_F_*PI*_	-1.628 (-2.999 – 0.257)	0.020	-1.540 (-2.891 – -0.189)	0.025
log_10_F_*RTI*_	1.465 (-0.683 – 3.613)	0.181	1.583 (-0.527 – 3.692)	0.141
log_10_F_*PI*−*m*_	-1.861 (-3.314 – -0.407)	0.012	-1.779 (-3.213 – -0.346)	0.015
log_10_F_*RTI*−*m*_	1.400 (-1.733 – 4.532)	0.381	1.356 (-1.727 – 4.439)	0.389

Linear regression analyses of the association between genotypic predictors and clinical parameters. Viral load values were log transformed and CD4 counts were square root transformed to approximate the normal distribution. For each of the genotypic predictors, two models including different sets of potential confounders were investigated. Model 1 included genotypic predictors for PR and RT, and estimated duration of infection (recent vs unknown duration). Model 2 additionally included age, gender, risk group and area of origin.

The analysis was subsequently extended by two additional approaches to further investigate the role of genetic determinants and to exclude a trend of TDR over calendar year as a confounding factor. In a first approach, we reverted major resistance mutations present in the viral sequences (log_10_F_*PI*−*m*_, log_10_F_*RTI*−*m*_) (Table 3). Second, patients with genotypic evidence of TDR were excluded from the analysis (Table 4). In both approaches, the estimated fitness under the PI selective pressure model remained significantly associated with viral load and CD4 count, while the association of the fitness under the RTI selective pressure model remained not significant. These results suggest that the correlations found are mainly attributable to polymorphic mutations in PR, that also confer a selective advantage under treatment selective pressure.

**Table 4:** Regression analysis to predict viral load and CD4 count in patients without evidence of TDR

	**Model 1**		**Model 2**	
**Factors**	**log VL (95% CI)**	**p-value**	**log VL (95% CI)**	**p-value**
log_10_F_*PI*_	0.298 (0.122 – 0.421)	0.000	0.269 (0.105 – 0.433)	0.001
log_10_F_*RTI*_	-0.090 (-0.324 – 0.144)	0.449	-0.219 (-0.583 – 0.144)	0.237
**Factors**	**sqrt CD4 (95% CI)**	**p-value**	**sqrt CD4 (95% CI)**	**p-value**
log_10_F_*PI*_	-2.038 (-3.556 – -0.521)	0.009	-1.947 (-3.446 – -0.448)	0.011
log_10_F_*RTI*_	1.220 (-2.145 – 4.585)	0.477	1.243 (-2.074 – 4.560)	0.462

Linear regression analyses of the association between genotypic predictors and clinical parameters. Patients that did show genotypic evidence of TDR were excluded from the analysis. For each of the genotypic predictors, two models including different sets of potential confounders were performed. Model 1 included genotypic predictors for PR and RT, and estimated duration of infection. Model 2 additionally included age, sex, risk group and area of origin.

### Determining the contribution of individual mutations at polymorphic positions

**Figure 1:**
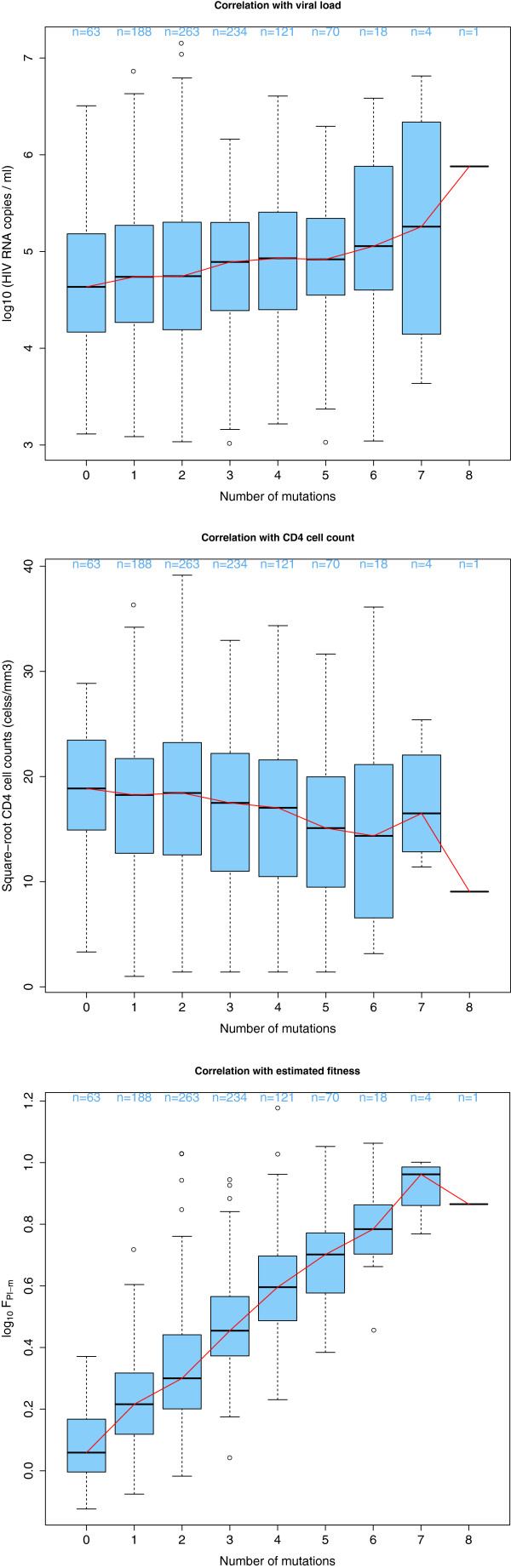
**Association between the number of compensatory mutations, estimated fitness and clinical parameters.** Compensatory mutations, polymorphic in subtype B protease and modeled by the fitness model F_*PI*_, are likely candidates to explain the observed association of viral fitness estimated under drug selective pressure with clinical parameters. For patients with no indications of acute infection and TDR (n = 962), the number of compensatory mutations (13V, 36I, 60E, 62V, 63P, 71V, 71T, 77I or 93L) in the protease sequence is calculated for each patient [21]. The following parameters are grouped by mutation count: log_10_ viral RNA copies/ml (1a), square-root transformed CD4 cell counts (1b) and increased estimated fitness for protease log_10_F_*PI*−*m*_(1c). The distribution of the respective parameter is shown for each group using boxplots. The horizontal line (bold) within the boxplot represents the median value, with box boundaries indicating the interquartile range. Upper and lower ends of striped lines denote the most extreme data point which is no more than 1.5 times the IQR range from the box. An increased mutation number significantly correlated with 1a) increased log_10_ viral RNA copies/ml (p-value < 0.01), 1b) decreased square-root transformed CD4 cell counts (p-value < 0.01) and 1c) increased estimated fitness for protease log_10_F_*PI*−*m*_(p-value < 0.01). A fitted line going through the median values (lowess smooth) is shown in red. The number of patients for each group is shown above each bin.

The fitness models were designed to predict fitness under drug selective pressure. In addition to well known mutations conferring (high-level) phenotypic drug resistance to protease inhibitors, mutations contributing to estimated fitness under the PR model included also known minor or accessory resistance mutations, either non-polymorphic or natural polymorphisms, and several other polymorphic positions (See Additional file 1: Fitness landscape). Univariate analysis with correction for multiple testing did not identify a single mutation that significantly affected viral load or CD4 cell count (data not shown). This suggested that combinatorial patterns of mutations in protease rather than single-mutation effects explained the observed associations, arguing in favor of epistatic interactions between mutations. Naturally occurring polymorphisms in protease (L10I/V, I13V, K20I/M/R, M36I, D60E, I62V, L63P, A71T/V, V77I and I93L) have previously been associated with therapy experience, and were also modeled by the fitness model F_*PI*_. We evaluated the correlation of the presence of these polymorphisms with viral load or CD4 count. Several of these compensatory mutations were highly polymorphic in the subtype B study population (Table 5). The distribution of these therapy-associated polymorphisms was similar in recently infected patients compared to patients with unknown duration of infection (data not shown). Figure 1 shows that a higher number of polymorphic mutations in protease was significantly associated (p-value < 0.01) with a higher viral load (1a), a lower CD4 count (1b), and a higher estimated fitness (log_10_F_*PI*−*m*_ where major PI resistance mutations had been reverted) (1c). No significant trend was observed for RT polymorphic mutations, in agreement with the lack of association of estimated fitness under the RT drug selective pressure fitness model and viral load or CD4 count (data not shown). These results further support the hypothesis that polymorphic amino acids significantly contributed to the observed associations between PR fitness estimated using a drug selective pressure fitness model and viremia or CD4 cell count in absence of treatment.

**Table 5:** Prevalence of polymorphic compensatory mutations in subtype B protease

**Mut**	**%**	**n**	**Mut**	**%**	**n**
10I	10.02	160	60E	9.2	147
10V	2.76	44	62V	32.75	523
13V	17.09	273	63P	58.42	933
20I	0.13	2	71T	9.58	153
20M	0.69	11	71V	7.2	115
20R	3.44	55	77I	32.19	514
36I	17.72	283	93L	42.45	678

The prevalence of known polymorphic compensatory mutations [21] in the subtype B study population is shown as percentages (%) and absolute count (n).

## Discussion

A substantial number of newly diagnosed HIV-1 patients is infected with a drug resistant virus, carrying the footprints of drug selective pressure [4-6]. Reversion of major resistance mutations in absence of therapy, both in treatment-experienced patients and in patients with transmitted drug resistance [3,5], illustrates that these mutations contribute in vivo to a decrease in viral fitness in absence of drug pressure. Considering that TDR is largely defined by non-polymorphic treatment-related mutations, mainly reflecting major drug resistance mutations [20], TDR has initially been speculated to result in lower set-point viral loads and higher CD4 cell counts, and consequently a slower disease progression [11]. While studies have reported that transmission of drug-resistant virus was associated with changes in initial viral load and CD4 counts in both seroconverters and chronically HIV-infected patients [11,22], other studies could not corroborate these findings [23,24]. Compensatory mutations often accompany major resistance mutations, selected to restore impaired intrinsic replication capacity [21]. We hypothesized that these accessory mutations could also increase viral fitness in absence of drug selective pressure or major drug resistance mutations. If true, TDR could potentially contribute to a higher viral load and a lower CD4 cell count in therapy naive patients, which is the opposite as previously speculated.

To investigate this hypothesis, we evaluated retrospectively the association of viral genotype with baseline viremia and CD4 cell count in a population of newly diagnosed treatment-naive HIV-1 patients from the SPREAD study [4,5]. We quantified the genetic variability with respect to its potential contribution to drug selective pressure using the most recent WHO surveillance drug resistance mutation list [20], a set of known compensatory mutations [21], and an vivo fitness landscape (FL) of drug selective pressure. In this study, viral load and CD4 cell count did not differ between patients with or without TDR, in agreement with earlier reports [23,24]. Evidence of TDR primarily consisted of single mutations, often 215 revertants [4,5], indicating that major resistance mutations had already reverted in the majority of the TDR patients, along with their deleterious effects on virus replication in absence of drug. FLs modeled the fitness contribution of a large number of mutations, including polymorphic compensatory mutations. We observed significantly higher viral loads and lower CD4 counts in patients infected with a virus deemed fitter by the PI drug selective pressure model, even after correcting for possible confounders. This association remained significant after the exclusion of TDR patients from the analysis or of fitness contributions of major drug resistance mutations. These findings suggest that amino acids at polymorphic positions in PR, more frequently observed in patients failing therapy, can increase in vivo fitness in absence of therapy and major resistance mutations. The correlation between the number of known polymorphic, compensatory mutations in PR [21] and changes in viral load and CD4 count supported the results of estimated fitness. Regression analyses showed relatively small correlation coefficients, indicating that observed variability in clinical parameters could only be partially explained, and emphasizing on a larger role of other host- and virus-related factors at play in absence of treatment. Viremia showed a stronger relationship with estimated fitness, compared to CD4 count, which would be consistent with the notion that viremia is a more direct outcome of the viral life cycle. Estimated fitness by the RTI drug selective pressure model did not significantly correlate with viral load and CD4 cell count. However, this fitness landscape model and the analyzed sequences included only the part of RT that is usually sequenced for drug resistance purposes (position 1 to 230), and therefore the full potential of treatment-related variability in RT remains to be further explored. The fact that the PR gene region is less conserved not only between different subtypes [25], but also within a single subtype [26], may indicate that purifying selection acts stronger on RT, suggesting it may be easier to find fitness differences in PR compared to RT.

In vitro studies have observed a wide range of HIV-1 replication capacity of *pol* genotypes both from recently and chronically infected treatment-naive patients, even after excluding viruses with genotypic evidence of drug resistance [17,27-30]. Patients infected with a virus showing lower in vitro *pol* replication capacity had significantly lower baseline HIV-1 RNA levels [27,28] and higher baseline CD4 cell counts. This association was independent of duration of infection and presence of drug resistance [17,28], and showed to be predictive for disease progression [28,30]. Although in vitro replication capacity is differently quantified than our modeled in vivo fitness, and neither is a direct measurement of in vivo fitness, these reports further suggest that improved replication capacity increases the virulence of HIV-1.

Importantly, although our study shows that compensatory mutations can increase in vivo fitness in absence of deleterious resistance mutations, we do not claim that this higher fitness was caused by transmitted drug resistance, as many compensatory mutations constitute natural genotypic variation in subtype B [12]. However, given that these mutations are frequently selected under drug selective pressure, our results do indicate the potential of transmitted drug resistance to impact on the natural history of HIV-1 at a population level. While still highly speculative, the observed correlation with CD4 count suggests in addition a worse clinical outcome in these therapy naive patients that harbor a fitter virus, whether or not the responsible polymorphisms were naturally present [12] or resulted from TDR. If ART selects in addition to antiviral resistance for more efficient HIV-1 enzymes, and such substitutions are being transmitted, then these alterations should be detectable at the epidemic level. With the current problem of transmitted drug resistance, we predict that polymorphisms improving virus fitness in both presence and absence of drugs may increase in prevalence over time. Furthermore, we speculate that TDR may be changing the HIV-1 epidemic and potentially pushing the virus to become more pathogenic. Additional investigation is warranted to confirm this proposed potential of TDR.

Our study suggests an intricate connection between HIV-1 natural diversity, protease plasticity, changes in viral fitness and potentially disease progression. While individual mutations, resistance related or polymorphic, have been studied in terms of enzyme activity, replicative capacity and drug susceptibility [13,14,31-33], systematic analyses are needed to better understand their combined effect through complex epistatic interactions as suggested in this manuscript. Similar combinatorial complexity may be anticipated for mutations in protease interacting with cleavage sites in the gag polyprotein [34], and CTL epitopes involved in viral escape from host selective pressure [35]. Empirical studies can further address difficulties in the interpretation of relative estimated fitness, which was originally conceived and validated in an environment of treatment pressure, and in the translation to clinical implications.

A criticism of our findings could be that if compensatory mutations increase in vivo fitness in absence of therapy, viruses with fitter protease should already have been naturally selected given that HIV-1 subtype B viruses have been circulating for decades in the human population. In this respect, it is important to discriminate within-host from between-host selective pressure. The within-host evolution in absence of drug selective pressure is dominated by immune selective pressure exerting a strong diversifying selection mainly on the immunodominant envelope [36]. The prevalence of particular polymorphisms in the infected human population is however dependent on the epidemic fitness of HIV, which is a very complex parameter and governed by between-host effects such as transmission efficiency and the number of new infections per infected individual. These effects depend not only on the level of the viral load, but also on the life expectancy of the transmitter (length of the asymptomatic phase) which may decrease with increasing viral load.

One could also argue that, since protease TDR is decreasing over calendar year [37], we observed the same trend in compensatory mutations such that those patients with more compensatory mutations were actually infected for a longer time. This confounding factor was taken into account by performing a separate analysis whereby TDR patients were excluded from the analysis. In this additional analysis, we still detected a significant association between presence of compensatory mutations in protease, higher viral load and lower CD4 count.

A major limitation of this study is the cross-sectional design, consecutive measurements of viral load and CD4 count are needed to better assess the impact of the reported mutations on disease progression. Indeed, CD4 cell count decline and viral load rise are associated with longer duration of infection and natural disease progression. Therefore, it can not be excluded that natural disease progression is associated with a rise in prevalence of these reported mutations, and what we observe is merely a consequence of this process. However, in our cohort, neither TDR, estimated fitness values nor the number of known compensatory mutations differed between recently and chronically infected patients. Second, the regression model was extended with a variable indicating whether a patient was recently infected or not, which did not change the results. We are also not aware of any literature indicating that the here reported mutations increase in prevalence during the natural course of disease progression, while if they did, this would still not invalidate our hypothesis that these mutations have a negative impact on disease progression. In that case, we could interpret the transmission of such mutations as a head start of the virus in the course of disease progression.

To end, with respect to an individual HIV-1 infected patient, resistance-associated virological failure severely limits ART options due to the persistence of acquired drug resistance and the existence of within-class cross-resistance. We now suggest that the consequences also reach beyond a single patient as high rates of resistance detected at therapy failure increase the probability of TDR [38], transferring genotypic footprints of adaptive evolution under drug pressure to newly infected hosts [4]. If our hypothesis is true, in the newly infected patient, this may not only affect therapy response, but also change the natural course of disease progression. The impact of fitter virus in drug naive patients on therapy effectiveness is currently unknown, though better replicating virus can lower the genetic barrier to resistance by facilitating resistance development and compromising the long term benefit of antiretroviral treatment both at the individual and the population level [39,40].

## Conclusions

It has previously been shown that in absence of treatment drug-resistant HIV-1 usually displays a lower replication capacity than wild-type virus due to the deleterious effect of major resistance mutations. These major resistance mutations tend to revert quickly after transmission to a new host, while compensatory mutations that restore enzymatic efficiency rather than confer antiviral resistance do not experience this selective pressure for reversion. Our results lead us to speculate that antiviral treatment pressures the virus to optimize its enzymes and is selecting for a more fit, and possibly also more virulent virus at population level. Increased availability of antiretroviral treatment and transmission of treatment-selected polymorphisms could have clinical implications both at the individual and the population level. These findings could provide additional complexity to the current and ongoing controversy on whether HIV-1 virulence is changing over time [41].

## Methods

### Ethics statement

Ethical requirements are fulfilled according to the procedure described in the EC contract. The procedure differs among the 32 countries in the network according to national legislation (the national reference laboratory and corresponding national coordinator for each country are listed in Additional file 3). Briefly, for each participating hospital or collection center, approval was obtained by the institutional medical ethical review committee. Additionally, a written informed consent was obtained for each patient. In countries where a mandatory surveillance system was already established, legally no informed consent was needed. All surveillance data were made anonymous and coded at national level.

### Study population

The European SPREAD project is a surveillance programme that prospectively collected representative data of HIV-1 infected individuals, newly diagnosed between September 2002 and December 2005 in 20 European countries (Austria, Belgium, Cyprus, Czech Republic, Germany, Denmark, Spain, Finland, Greece, Ireland, Italy, Luxembourg, the Netherlands, Norway, Poland, Portugal, Sweden, Slovenia, Slovakia and Serbia) and Israel. Previous reports of the SPREAD programme focused on the analyses of transmitted drug resistance and HIV-1 subtype distribution in Europe [5,6]. A standardized sampling strategy was designed by the epidemiology expert group of the SPREAD programme to ensure representative sampling in all countries. Patients were eligible if they had not been submitted to antiretroviral therapy by the time of sampling and if they were at least 18 years old. Furthermore, the first available sample obtained within 6 months of HIV-1 diagnosis was used, with a predefined viral load inclusion threshold above 1000 HIV-1 RNA copies/ml, since that was the threshold defined necessary at the time for reliable genotype testing. The few genotypes associated to a sample with a lower viral load were therefore considered a source of bias and excluded from all SPREAD studies. Epidemiological, clinical and behavioral data were collected using a standardized questionnaire. Patients were defined as recently infected if they had documented negative or indeterminate HIV-1 serological results up to 12 months prior to confirmation of diagnosis. The remaining newly diagnosed patients were classified as those with undefined duration of infection. In line with other clinical cohort studies, HIV-RNA viral load levels and CD4 cell counts used in this study were determined locally using assays validated for clinical use. Genotypic analysis was decentralized by local laboratories using either in-house methods or commercially available genotypic resistance testing kits, and the raw nucleotide sequence data were used in the current study. All laboratories participated in a continuous blinded quality control programme to verify the quality of the data.

### HIV-1 fitness landscape

HIV-1 adaptation to drug selective pressure can be modeled by observing viral evolution in patients at treatment failure. When the same mutation is independently fixed in multiple patients under selective pressure of the same treatment, it may be assumed that this convergent evolution indicates an increased fitness of the mutant virus under that treatment. Since a synergistic interaction between two mutations is expected to lead to a different observed prevalence of one mutation depending on the presence of the other, observed associations in prevalence may indicate epistatic fitness interactions between these mutations [42]. Hence, a fitness function of HIV-1 can be learned based on the difference in prevalence of mutations in viral sequences from treatment-experienced patients compared with untreated patients. Previously, we developed an evolutionary framework to reconstruct such an in vivo fitness landscape (FL), by quantitatively estimating the influence of mutations and mutation patterns on HIV-1 fitness during treatment, solely as a function of the genotypic sequence. This estimated fitness under treatment pressure reflects the combined effect of drug resistance and replication capacity. Briefly, a fitness function was constructed following a two-step process (see [19] and Additional file 1 for a detailed description of the methodology). First, conditional dependencies between mutations were identified using a probabilistic model that efficiently summarized observed correlations between mutations. For each mutational pattern that was modeled, the fitness function specified a separate fitness contribution. Secondly, the selective advantage of each incorporated mutation interaction was quantified in an iterative step, where the viral evolution under treatment was simulated using the fitness landscape. Differences between predicted and observed prevalence of the included mutation patterns were measured and minimized, and fitness values were optimized until convergence wasreached.

We previously constructed FLs for specific protease inhibitors (PI) [19], or for specific combinations of reverse transcriptase inhibitors (RTI) [43] and have shown that fitness models can significantly predict therapy response in vivo [44]. For the current study, we constructed two generic fitness functions, one modeling the selective pressure on protease (PR) by any protease inhibitor (PI), and one modeling the selective pressure on reverse transcriptase (RT) by any reverse transcriptase inhibitor (RTI) (See Additional file 1). To build the fitness functions, data were pooled from Portugal, Belgium and the Stanford Drug Resistance database only using data independent from data within the SPREAD program. A total of 3751 sequences were from patients treated with one or more PIs and 8328 sequences from PI naive patients. A total set of 1736 sequences were from patients treated with one or more RTIs and 3769 sequences from RTI naive patients. The models not only included (major) drug resistance mutations, but also any polymorphism with a prevalence of >1% (PI) or >3% (RTI) in the respective treated population. The PR fitness landscape FL_*PI*_included 104 mutations and contained 898 different mutation interactions. The RT landscape FL_*RTI*_included 112 mutations and modeled 1172 possible mutation patterns. (See Additional file 1). The two fitness landscapes were scaled to a fitness of 1 for HIV-1 subtype B reference strain HXB2. For any given sequence, the fitness landscape computes a single fitness value that represents the relative fitness compared to HXB2.

### Genotypic predictions

Viral subtype was assessed on the combined PRO-RT sequence using the REGA HIV-1 subtyping tool V2 [45]. Evidence of transmitted drug resistance was defined as the presence of at least one surveillance drug-resistance mutation [20]: 23I, 24I, 30N, 32I, 46IL, 47AV, 48MV, 50VL, 53LY, 54AMLSTV, 73ACST, 76V, 82ACFLMST, 83D, 84ACV, 85V, 88DS or 90M in PR; and 41L, 65R, 67EGN, 69insD, 70ER, 74IV, 75AMST, 77L, 100I, 101EP, 103NS, 106AM, 115F, 116Y, 151M, 179F, 181CIV, 184IV, 188CHL, 190AES, 210W, 215CDEFISVY, 219ENRQ, 225H or 230L in RT. Major drug resistance mutations were defined according to the International AIDS Society USA (IAS-USA) [19]. For PR, these were 30N, 32I, 33F, 43T, 46L, 47V, 48V, 50LV, 54LMV, 58E, 74SP, 76V, 82AFT, 84V, 88DS, and 90M. For RT these were: 41L, 44D, 62V, 65R, 67N, 70R, 74IV, 75I, 77L, 100I, 103N, 106AM, 108I, 115F, 116Y, 118I, 151M, 181C, 188CHL, 190AS, 210W, 215FY, 219EQ and 225H. Compensatory drug resistance mutations, all polymorphic variants in wild-type virus, were defined according to Shafer et al. [21]. For PR, these were 13V, 36I, 60E, 62V, 63P, 71V, 71T, 77I and 93L. For RT, polymorphic accessory mutations were 98S, 101R, 101Q, 106I, 138A, 179I and 238R. For a given genotype in the study population, fitness values under PI (F_*PI*_) and RTI (F_*RTI*_) selective pressure, were computed using the models FL_*PI*_and FL_*RTI*_respectively. Such a FL scores a higher fitness to both major resistance mutations and compensatory mutations, while in absence of drugs our hypothesis would assign a negative impact of the major resistance mutation and a positive impact of the compensatory mutation. Therefore, the effect of major resistance mutations was excluded in two ways, which allowed to capture the contribution of the compensatory mutations. A first approach constituted the exclusion of the subset of patients with indications of TDR [20] from the analysis. In a second approach, an additional fitness value (F_*PI*−*m*_ or F_*RTI*−*m*_) was computed for sequences that displayed major resistance mutations, by reverting the major resistance mutation to the corresponding wild-type amino acid.

### Statistical analysis

TDR prevalence values were calculated with a 95% Wilson score confidence interval based on the binomial distribution. A log_10_ transformation of viral load and a square root transformation of CD4 count were applied in order to obtain a normal distribution of variance. For continuous variables, comparisons between means were conducted by using a t-test or a Mann Whitney U test. For categorical variables, comparisons between proportions were conducted by using the contingency-table *χ*_2_ test. Multivariate linear models were constructed to determine the ability of the genotypic factors to predict CD4+ T cell counts (square root transformed) and plasma HIV-1 RNA levels (log_10_ transformed). Two multivariable models were fitted. A first model included, besides genotypic predictors, a variable indicating evidence of acute infection in order to take into account the bias of patients that had not reached the set-point viral load [46,47]. In a second model, we adjusted for a number of additional potential confounders listed in Table 1. All analyses were performed using the statistical software R (version 2.12.0).

## Competing interests

The authors declare that they have no competing interests.

## Authors’ contributions

KT and KD designed and implemented the analysis. KT, KD, JV and PL performed the analyses. KT and AMV drafted the manuscript. DvdV, JA, BA, CB, MB, RJC, BC, SC, ZG,OH, AH, KK, LGK, CK, CN, DP, MP, EPS, CR, LR, KL, JCS, RS, AS, DS, MS, DS, KVL, AMJW and CABB contributed clinical and virological data. All co-authors contributed to the interpretation of the results. All authors have read and approved the final manuscript.

## Supplementary Material

Additional file 1 **Modeling a fitness landscape of HIV-1 under drug selective pressure.** An overview of the estimation procedure including a list of mutations included in the protease or reverse transcriptase fitness function. Click here for file

Additional file 2 **Prevalence of fitness function mutations in drug naive patients.** For each mutation that is modeled by the fitness landscapes, the prevalence in the study population of subtype-B infected recently diagnosed drug naive patients is shown as percentage and absolute count. Click here for file

Additional file 3 **List of participating centers.** List of participating national reference laboratories in the network. Click here for file

## References

[B1] Vandamme AM Van Laethem K De Clercq E Managing resistance to anti-HIV drugs: an important consideration for effective disease management Drugs 1999 57 337 361 10.2165/00003495-199957030-00006 10193687

[B2] Martinez-Picado J Martinez MA HIV-1 reverse transcriptase inhibitor resistance mutations and fitness: a view from the clinic and ex vivo Virus Res 2008 134 104 123 10.1016/j.virusres.2007.12.021 18289713

[B3] Verhofstede C Wanzeele FV Van Der Gucht B De Cabooter N Plum J Interruption of reverse transcriptase inhibitors or a switch from reverse transcriptase to protease inhibitors resulted in a fast reappearance of virus strains with a reverse transcriptase inhibitor-sensitive genotype AIDS 1999 13 2541 2546 10.1097/00002030-199912240-00007 10630523

[B4] Wensing AM van de Vijver DA Angarano G Asjö B Balotta C Boeri E Camacho R Chaix ML Costagliola D De Luca A Derdelinckx I Grossman Z Hamouda O Hatzakis A Hemmer R Hoepelman A Horban A Korn K Kücherer C Leitner T Loveday C MacRae E Maljkovic I de Mendoza C Meyer L Nielsen C Op de Coul EL Ormaasen V Paraskevis D Perrin L Prevalence of drug-resistant HIV-1 variants in untreated individuals in Europe: implications for clinical management J Infect Dis 2005 192 958 966 10.1086/432916 16107947

[B5] Wensing A Vercauteren J van de Vijver D Albert J Åsjö B Claudia B Camacho R Coughlan S Grossman Z Horban A Kücherer C Nielsen C Paraskevis D Loke W Poggensee G Riva C Ruiz L Schmit J Schuurman R Salminen M Sonnerborg A Stanojevic M Struck D Vandamme A Boucher CA Transmission of drug-resistant HIV-1 in Europe remains limited to single classes AIDS 2008 22 625 635 18317004 10.1097/QAD.0b013e3282f5e062

[B6] Vercauteren J Wensing AM van de Vijver DA Albert J Balotta C Hamouda O Kücherer C Struck D Schmit JC Asjö B Bruckova M Camacho RJ Clotet B Coughlan S Grossman Z Horban A Korn K Kostrikis L Nielsen C Paraskevis D Poljak M Puchhammer-Stöckl E Riva C Ruiz L Salminen M Schuurman R Sonnerborg A Stanekova D Stanojevic M Vandamme AM Transmission of drug-resistant HIV-1 is stabilizing in Europe J Infect Dis 2009 200 1503 1508 10.1086/644505 19835478

[B7] Simon V Padte N Murray D Vanderhoeven J Wrin T Parkin N Di Mascio M Markowitz M Infectivity and replication capacity of drug-resistant human immunodeficiency virus type 1 variants isolated during primary infection J Virol 2003 77 7736 7745 10.1128/JVI.77.14.7736-7745.2003 12829813 PMC161921

[B8] Bezemer D de Ronde A Prins M Porter K Gifford R Pillay D Masquelier B Fleury H Dabis F Back N Jurriaans S van der Hoek L Evolution of transmitted HIV-1 with drug-resistance mutations in the absence of therapy: effects on CD4+ T-cell count and HIV-1 RNA load Antivir Ther (Lond) 2006 11 173 178 16640098

[B9] van Maarseveen NM de Jong D Boucher CA Nijhuis M An increase in viral replicative capacity drives the evolution of protease inhibitor-resistant human immunodeficiency virus type 1 in the absence of drugs J Acquir Immune Defic Syndr 2006 42 162 168 10.1097/01.qai.0000219787.65915.56 16645546

[B10] Cong ME Heneine W García-Lerma JG The fitness cost of mutations associated with human immunodeficiency virus type 1 drug resistance is modulated by mutational interactions J Virol 2007 81 3037 3041 10.1128/JVI.02712-06 17192300 PMC1865994

[B11] Grant RM Hecht FM Warmerdam M Liu L Liegler T Petropoulos CJ Hellmann NS Chesney M Busch MP Kahn JO Time trends in primary HIV-1 drug resistance among recently infected persons JAMA 2002 288 181 188 10.1001/jama.288.2.181 12095382

[B12] Kozal MJ Shah N Shen N Yang R Fucini R Merigan TC Richman DD Morris D Hubbell E Chee M Gingeras TR Extensive polymorphisms observed in HIV-1 clade B protease gene using high-density oligonucleotide arrays Nat Med 1996 2 753 759 10.1038/nm0796-753 8673920

[B13] Holguin A Sune C Hamy F Soriano V Klimkait T Natural polymorphisms in the protease gene modulate the replicative capacity of non-B HIV-1 variants in the absence of drug pressure J Clin Virol 2006 36 264 271 10.1016/j.jcv.2006.05.001 16765636

[B14] Martinez-Picado J Savara AV Shi L Sutton L D’Aquila RT Fitness of human immunodeficiency virus type 1 protease inhibitor-selected single mutants Virology 2000 275 318 322 10.1006/viro.2000.0527 10998332

[B15] Alizon S von Wyl V Stadler T Kouyos RD Yerly S Hirschel B Boni J Shah C Klimkait T Furrer H Rauch A Vernazza PL Bernasconi E Battegay M Burgisser P Telenti A Gunthard HF Bonhoeffer S Phylogenetic approach reveals that virus genotype largely determines HIV set-point viral load PLoS Pathog 2010 6 e1001123 10.1371/journal.ppat.1001123 20941398 PMC2947993

[B16] Tang J Tang S Lobashevsky E Zulu I Aldrovandi G Allen S Kaslow RA HLA allele sharing and HIV type 1 viremia in seroconverting Zambians with known transmitting partners AIDS Res Hum Retroviruses 2004 20 19 25 10.1089/088922204322749468 15000695

[B17] Barbour JD Hecht FM Wrin T Segal MR Ramstead CA Liegler TJ Busch MP Petropoulos CJ Hellmann NS Kahn JO Grant RM Higher CD4+ T cell counts associated with low viral pol replication capacity among treatment-naive adults in early HIV-1 infection J Infect Dis 2004 190 251 256 10.1086/422036 15216458

[B18] Baeten JM Chohan B Lavreys L Chohan V McClelland RS Certain L Mandaliya K Jaoko W Overbaugh J HIV-1 subtype D infection is associated with faster disease progression than subtype A in spite of similar plasma HIV-1 loads J Infect Dis 2007 195 1177 1180 10.1086/512682 17357054

[B19] Deforche K Camacho R Van Laethem K Lemey P Rambaut A Moreau Y Vandamme AM Estimation of an in vivo fitness landscape experienced by HIV-1 under drug selective pressure useful for prediction of drug resistance evolution during treatment Bioinformatics 2008 24 34 41 10.1093/bioinformatics/btm540 18024973

[B20] Bennett DE Camacho RJ Otelea D Kuritzkes DR Fleury H Kiuchi M Heneine W Kantor R Jordan MR Schapiro JM Vandamme AM Sandstrom P Boucher CA van de Vijver D Rhee SY Liu TF Pillay D Shafer RW Drug resistance mutations for surveillance of transmitted HIV-1 drug-resistance 2009, update PLoS ONE 2009 4 e4724 10.1371/journal.pone.0004724 19266092 PMC2648874

[B21] Shafer RW Schapiro JM HIV-1 drug resistance mutations: an updated framework for the second decade of HAART AIDS Rev 2008 10 67 84 18615118 PMC2547476

[B22] Peuchant O Thiebaut R Capdepont S Lavignolle-Aurillac V Neau D Morlat P Dabis F Fleury H Masquelier B Dabis F Chene G Dabis F Lawson-Ayayi S Thiebaut R Winnock M Bonarek M Bonnal F Bonnet F Bernard N Caubet O Caunegre L Cazanave C Ceccaldi J Couzigou P Dauchy FA De La Taille C De Witte MC Dupon M Duffau P Dutronc H Transmission of HIV-1 minority-resistant variants and response to first-line antiretroviral therapy AIDS 2008 22 1417 1423 10.1097/QAD.0b013e3283034953 18614864

[B23] Bhaskaran K Pillay D Walker AS Fisher M Hawkins D Gilson R McLean K Porter K Do patients who are infected with drug-resistant HIV have a different CD4 cell decline after seroconversion? An exploratory analysis in the UK Register of HIV Seroconverters AIDS 2004 18 1471 1473 10.1097/01.aids.0000131341.45795.33 15199326

[B24] Jakobsen MR Tolstrup M Søgaard OS Jørgensen LB Gorry PR Laursen A Ostergaard L Transmission of HIV-1 drug-resistant variants: prevalence and effect on treatment outcome Clin Infect Dis 2010 50 566 573 10.1086/650001 20085464

[B25] Vergne L Peeters M Mpoudi-Ngole E Bourgeois A Liegeois F Toure-Kane C Mboup S Mulanga-Kabeya C Saman E Jourdan J Reynes J Delaporte E Genetic diversity of protease and reverse transcriptase sequences in non-subtype-B human immunodeficiency virus type 1 strains: evidence of many minor drug resistance mutations in treatment-naive patients J Clin Microbiol 2000 38 3919 3925 11060045 10.1128/jcm.38.11.3919-3925.2000 PMC87518

[B26] Turner D Roldan A Brenner B Moisi D Routy JP Wainberg MA Variability in the PR and RT genes of HIV-1 isolated from recently infected subjects Antivir Chem Chemother 2004 15 255 259 15535047 10.1177/095632020401500504

[B27] Campbell TB Schneider K Wrin T Petropoulos CJ Connick E Relationship between in vitro human immunodeficiency virus type 1 replication rate and virus load in plasma J Virol 2003 77 12105 12112 10.1128/JVI.77.22.12105-12112.2003 14581547 PMC253754

[B28] Daar ES Kesler KL Wrin T Petropoulo CJ Bates M Lail A Hellmann NS Gomperts E Donfield S HIV-1 pol replication capacity predicts disease progression AIDS 2005 19 871 877 10.1097/01.aids.0000171400.15619.e1 15905667

[B29] De Luca A Weidler J Di Giambenedetto S Coakley E Cingolani A Bates M Lie Y Pesano R Cauda R Schapiro J Association of HIV-1 replication capacity with treatment outcomes in patients with virologic treatment failure J Acquir Immune Defic Syndr 2007 45 411 417 10.1097/QAI.0b013e318074f008 17554216

[B30] Goetz MB Leduc R Wyman N Kostman JR Labriola AM Lie Y Weidler J Coakley E Bates M Luskin-Hawk R HIV replication capacity is an independent predictor of disease progression in persons with untreated chronic HIV infection J Acquir Immune Defic Syndr 2010 53 472 479 10.1097/QAI.0b013e3181cae480 20032783 PMC2837106

[B31] Santos AF Abecasis AB Vandamme AM Camacho RJ Soares MA Discordant genotypic interpretation and phenotypic role of protease mutations in HIV-1 subtypes B and G J Antimicrob Chemother 2009 63 3 593 599 10.1093/jac/dkn526 19136678

[B32] Santos AF Tebit DM Lalonde MS Abecasis AB Ratcliff A Camacho RJ Diaz RS Herchenroder O Soares MA Arts EJ Effect of natural polymorphisms in the HIV-1 CRF02_AG protease on protease inhibitor hypersusceptibility Antimicrob Agents Chemother 2012 56 5 2719 2725 10.1128/AAC.06079-11 22330918 PMC3346603

[B33] Henderson GJ Lee SK Irlbeck DM Harris J Kline M Pollom E Parkin N Swanstrom R Interplay between single resistance-associated mutations in the HIV-1 protease and viral infectivity, protease activity, and inhibitor sensitivity Antimicrob Agents Chemother 2012 56 2 623 633 10.1128/AAC.05549-11 22083488 PMC3264268

[B34] Dam E Quercia R Glass B Descamps D Launay O Duval X Krausslich HG Hance AJ Clavel F Gag mutations strongly contribute to HIV-1 resistance to protease inhibitors in highly drug-experienced patients besides compensating for fitness loss PLoS Pathog 2009 5 3 e1000345 10.1371/journal.ppat.1000345 19300491 PMC2652074

[B35] Brockman MA Brumme ZL Brumme CJ Miura T Sela J Rosato PC Kadie CM Carlson JM Markle TJ Streeck H Kelleher AD Markowitz M Jessen H Rosenberg E Altfeld M Harrigan PR Heckerman D Walker BD Allen TM Early selection in Gag by protective HLA alleles contributes to reduced HIV-1 replication capacity that may be largely compensated for in chronic infection J Virol 2010 84 22 11937 11949 10.1128/JVI.01086-10 20810731 PMC2977869

[B36] Lemey P Rambaut A Pybus OG HIV evolutionary dynamics within and among hosts AIDS Rev 2006 8 125 140 17078483

[B37] Vercauteren J Deforche K Theys K Debruyne M Duque LM Peres S Carvalho AP Mansinho K Vandamme AM Camacho R The incidence of multidrug and full class resistance in HIV-1 infected patients is decreasing over time (2001-2006) in Portugal Retrovirology 2008 5 12 10.1186/1742-4690-5-12 18241328 PMC2265747

[B38] Paredes R Clotet B Clinical management of HIV-1 resistance Antiviral Res 2010 85 245 265 10.1016/j.antiviral.2009.09.015 19808056

[B39] Theys K Deforche K Beheydt G Moreau Y van Laethem K Lemey P Camacho RJ Rhee SY Shafer RW Van Wijngaerden E Vandamme AM Estimating the individualized HIV-1 genetic barrier to resistance using a nelfinavir fitness landscape BMC Bioinformatics 2010 11 409 10.1186/1471-2105-11-409 20682040 PMC2921410

[B40] Perno CF Cozzi-Lepri A Balotta C Forbici F Violin M Bertoli A Facchi G Pezzotti P Cadeo G Tositti G Pasquinucci S Pauluzzi S Scalzini A Salassa B Vincenti A Phillips AN Dianzani F Appice A Angarano G Monno L Ippolito G Moroni M d’ Arminio Monforte A Secondary mutations in the protease region of human immunodeficiency virus and virologic failure in drug-naive patients treated with protease inhibitor-based therapy J Infect Dis 2001 184 983 991 10.1086/323604 11574912

[B41] Herbeck JT Muller V Maust BS Ledergerber B Torti C Di Giambenedetto S Gras L Gunthard HF Jacobson LP Mullins JI Gottlieb GS Is the virulence of HIV changing? A meta-analysis of trends in prognostic markers of HIV disease progression and transmission AIDS 2012 26 193 205 10.1097/QAD.0b013e32834db418 22089381 PMC3597098

[B42] Deforche K Silander T Camacho R Grossman Z Soares MA Van Laethem K Kantor R Moreau Y Vandamme AM Analysis of HIV-1 pol sequences using Bayesian networks: implications for drug resistance Bioinformatics 2006 22 2975 2979 10.1093/bioinformatics/btl508 17021157

[B43] Theys K Deforche K Libin P Camacho RJ Van Laethem K Vandamme AM Resistance pathways of human immunodeficiency virus type 1 against the combination of zidovudine and lamivudine J Gen Virol 2010 91 1898 1908 10.1099/vir.0.022657-0 20410311

[B44] Deforche K Cozzi-Lepri A Theys K Clotet B Camacho RJ Kjaer J Van Laethem K Phillips A Moreau Y Lundgren JD Vandamme AM Modelled in vivo HIV fitness under drug selective pressure and estimated genetic barrier towards resistance are predictive for virological response Antivir Ther (Lond) 2008 13 399 407 18572753

[B45] de Oliveira T Deforche K Cassol S Salminen M Paraskevis D Seebregts C Snoeck J van Rensburg EJ Wensing AM van de Vijver DA Boucher CA Camacho R Vandamme AM An automated genotyping system for analysis of HIV-1 and other microbial sequences Bioinformatics 2005 21 3797 3800 10.1093/bioinformatics/bti607 16076886

[B46] Boutwell CL Rolland MM Herbeck JT Mullins JI Allen TM Viral evolution and escape during acute HIV-1 infection J Infect Dis 2010 202 Suppl 2 S309 S314 20846038 10.1086/655653 PMC2945609

[B47] Langford S Ananworanich J Cooper D Predictors of disease progression in HIV infection: a review AIDS Res Ther 2007 4 11 10.1186/1742-6405-4-11 17502001 PMC1887539

