# Supplementary material for: Treatment-associated polymorphisms in protease are significantly associated with higher viral load and lower CD4 count in newly diagnosed drug-naive HIV-1 infected patients
Source: Retrovirology. 2012 Oct 3;9:81. doi: 10.1186/1742-4690-9-81 (PMC3487874; doi:10.1186/1742-4690-9-81)
Supplement: Additional file 2 — Prevalence of fitness function mutations in drug naive patients. For each mutation that is modeled by the fitness landscapes, the prevalence in the study population of subtype-B infected recently diagnosed drug naive patients is shown as percentage and absolute count. [file 1742-4690-9-81-S2.pdf]

Additional file 2

For the different mutations associated with the fitness models, the prevalence (percentage (%) and absolute number (n)) of these mutations in the study population of drug naive patients are shown for the subtype B study dataset (N= 1599). Mutations indicated in bold are considered in this study as major resistance mutations.

Prevalence of protease mutations

Mut	%	n	Mut	%	n	Mut	%	n	Mut	%	n
10I	10.02	160	**33F**	0.13	2	57K	14.03	224	71T	9.58	153
10F	0.5	8	33V	6.01	96	58E	0.44	7	71V	7.2	115
10V	2.76	44	35D	32.31	516	60E	9.2	147	72L	0.31	5
12A	5.57	89	35N	0.25	4	61E	3.51	56	72T	4.88	78
12S	4.7	75	35G	0.13	2	61H	0.88	14	72V	7.58	121
12P	3.69	59	36L	1.31	21	61N	0.56	9	72M	1.25	20
13V	17.09	273	36I	17.72	283	62V	32.75	523	**73S**	0.13	2
14R	14.4	230	36V	0.31	5	63A	5.57	89	74A	0.19	3
15V	19.04	304	37S	19.16	306	63T	6.26	100	74S	0.5	8
16E	4.01	64	37A	2.88	46	63H	2.63	42	77I	32.19	514
17E	0.75	12	37T	4.38	70	63Q	2.94	47	**82A**	0.56	9
18H	0.56	9	37H	2.07	33	63C	1.06	17	82I	2	32
19T	0.63	10	37E	3.63	58	63V	1	16	82F	0.13	2
19Q	1.19	19	37D	13.84	221	63P	58.42	933	**82T**	0	0
19I	9.52	152	39S	3.38	54	63S	0	0	**84V**	0.13	2
19V	1.13	18	41K	31	495	64L	6.83	109	85V	0.31	5
19P	0.06	1	43R	3.94	63	64V	14.4	230	**88D**	0.19	3
20R	3.44	55	**43T**	0.38	6	64M	2.19	35	**88S**	0	0
20I	0.13	2	45R	2.32	37	65D	4.76	76	89I	0.06	1
20T	0.13	2	**46L**	0.88	14	66F	0	0	89V	0	0
20V	0	0	**46I**	0.44	7	69R	0.94	15	89M	2	32
20M	0.69	11	**47V**	0.06	1	69Y	1.57	25	**90M**	0.75	12
**24I**	0.19	3	**48V**	0	0	69K	2.25	36	92R	0.31	5
**30N**	0.25	4	**53L**	0	0	69Q	5.01	80	92K	1.06	17
**32I**	0.06	1	**54V**	0.5	8	70R	1.5	24	93L	42.45	678
33I	0.75	12	55R	0.63	10	71I	0.19	3	95F	0	0

Prevalence of reverse transcriptase mutations

Mut	%	n	Mut	%	n	Mut	%	n	Mut	%	n
6D	6.01	96	69N	0.63	10	135L	1.44	23	**190A**	0.25	4
11T	1.13	18	69D	0.25	4	135T	33	527	196E	14.78	236
20R	9.89	158	**70R**	0.5	8	135V	6.7	107	200A	19.91	318
21I	1.94	31	**74I**	0.06	1	138A	2.69	43	200E	2	32
28K	0.94	15	**74V**	0.06	1	142T	4.2	67	200I	9.77	156
35L	1.82	29	83K	20.41	326	142V	9.33	149	202V	10.46	167
35T	5.95	95	90I	2.19	35	**151M**	0.19	3	203D	1.06	17
35M	3.51	56	**98G**	0.19	3	158S	2.5	40	203K	0.13	2
35I	13.71	219	98S	8.02	128	162C	19.04	304	207D	2.5	40
36A	1.13	18	**100I**	0.06	1	162Y	3.76	60	207G	0.69	11
39A	5.07	81	101Q	1	16	162A	5.7	91	207A	2.07	33
39E	0.13	2	**101E**	0.19	3	165I	5.01	80	207E	15.72	251
39K	0.63	10	102Q	3.63	58	166R	9.46	151	207K	3.57	57
40D	1.69	27	102R	2.07	33	169D	6.95	111	208Y	0.38	6
**41L**	1.63	26	**103N**	1.57	25	173T	0.38	6	**210W**	0.38	6
43Q	0.06	1	103R	1.31	21	173A	0.13	2	211R	39.82	636
43E	0.31	5	104R	2.13	34	173S	0.06	1	211S	2	32
43R	0.56	9	106I	2.44	39	173I	0.06	1	214L	15.84	253
44D	0.19	3	**108I**	0.69	11	173R	2.13	34	**215F**	0.19	3
48T	2.44	39	**118I**	2.63	42	174R	1.75	28	**215Y**	0.25	4
49R	7.08	113	121Y	2.44	39	174K	2.19	35	215S	1.06	17
60I	15.09	241	121H	3.94	63	177E	21.29	340	218E	0	0
**62V**	0.38	6	122P	3.38	54	178M	8.58	137	219N	0.06	1
64R	2	32	122K	68.94	1101	178L	7.2	115	**219Q**	0.63	10
65R	0.06	1	123G	1.5	24	179I	3.32	53	**219E**	0	0
67G	0.06	1	123N	4.51	72	**181C**	0.44	7	221Y	0.13	2
**67N**	0.38	6	123E	27.11	433	**184V**	0.25	4	228R	0.06	1
68G	5.32	85	123S	1.69	27	**188L**	0.06	1	228H	0.5	8
