# Supplementary material for: Treatment-associated polymorphisms in protease are significantly associated with higher viral load and lower CD4 count in newly diagnosed drug-naive HIV-1 infected patients
Source: Retrovirology. 2012 Oct 3;9:81. doi: 10.1186/1742-4690-9-81 (PMC3487874; doi:10.1186/1742-4690-9-81)
Supplement: Additional file 3 — List of participating centers. List of participating national reference laboratories in the network. [file 1742-4690-9-81-S3.pdf]

EuropeHIVResistance Project

Project Coordinator:

Dr. Charles Boucher
Department of Virology University Medical Center Utrecht
Heidelberglaan 100, 3584 CX Utrecht
Tel: +31 30 250 6526
Fax: +31 30 250 5426
E-mail: c.boucher@umcutrecht.nl

Project website: **www.europehivresistance.com**

List of participating national reference laboratories in the network:

(Countries indicated by ** provided data for the study described in the manuscript)

1. Institute of Virology, Medical University Vienna, Austria **
National Coordinator: Dr. E. Puchhammer-Stöckl

2. AIDS Reference Laboratory, REGA Institute and University Hospitals, Leuven, Belgium **
National Coordinator: Prof. Dr. A.M. Vandamme

3. National Center of Infectious and Parasitic Diseases, Department of Virology, Sofia, Bulgary
National Coordinator: Dr. D. Beshkov

4. Department of Biological Sciences, University of Cyprus , Nicosia, Cyprus **
National Coordinator: Prof. Dr. L.G. Kostrikis

5. National Reference Laboratory on AIDS, National Institute of Public Health, Prague, Czech Republic **
National Coordinator: Dr. Marek Linka

6. Department of Virology, Division of Diagnostics, Statens Serum Institute, Copenhagen, Denmark **
National Coordinator: Dr. C. Nielsen

7. West-Tallinn Central Hospital Centre for Infectious Diseases, Tallinn, Estonia
National Coordinator: Dr. K. Zilmer

8. HIV-Laboratory, Department of Infectious Disease Epidemiology,National Public Health Institute, Helsinki, Finland **
National Coordinator: Dr. Kirsi Liitsola

9. Bichat Hospital, Virology Laboratory, Paris, France
National Coordinator: Prof. dr. F. Brun-Vezinet

10. Department of Infectious Disease Epidemiology, Robert Koch Institute (RKI), Berlin, Germany **
National Coordinator: Dr. O. Hamouda

11. Department of Hygiene and Epidemiology, Hellenic Scientific Society for the Study of AIDS, Athens, Greece **
National Coordinator: Dr. A. Hatzakis

12. National Center for Epidemiology, Microbiological Research Group, Budapest, Hungary
National Coordinator: Dr. J. Minarovits

13. Department of Medical Microbiology, University College Dublin, Dublin, Ireland **
National Coordinator: Prof. dr. W. Hall

14. Sheba Medical Center & School of Public Health, Tel Aviv University, Tel Aviv, Israel **
National Coordinator: Dr. Z. Grossman

15. Institute of Infectious and Tropical Diseases, University of Milan, Milan **
National Coordinator: Prof. dr. C. Balotta

16. Infectology Center of Latvia, Riga, Latvia
National Coordinator: Dr. B. Rozentale

17. Laboratory department, Lithuanian AIDS centre, Vilnius, Lithuania
National Coordinator: Dr. A. Griskevicius

18. Retrovirology Laboratory, Centre de recherche Public – Sante, Strassen, Luxembourg **
National Coordinator: Dr. J.C. Schmit

19. Eijkman-Winkler Institute, Department of Virology, University Medical Center, Utrecht, The Netherlands
National Coordinator: Dr. C. Boucher
National Coordinator: Dr. A. Wensing

20. Center for Research in Virology, University of Bergen, Bergen, Norway **
National Coordinator: Dr. B. Asjö

21. Center for Diagnosis & Therapy Warsaw, Hospital for Infectious Diseases, Warszaw, Poland **
National Coordinator: Prof. A. Horban

22. Laboratório de Biologia Molecular, Serviço de Medicina Transfusional, Hospital Egas Moniz, Lisbon, Portugal **
National Coordinator: Dr. R. Camacho

23. "Prof. Dr. Matei Bals" Institute for Infectious Diseases, Bucharest, Romania
National Coordinator: Dr. A. Streinu-Cercel

24. Federal State Institution Central Research Institute of Epidemiology, Federal AIDS Center, Moscow, Russia
National Coordinator: Prof. V. Pokrovsky

25. Institute of Microbiology and Immunology, University of Belgrade School of Medicine, Belgrade, Serbia Montenegro **
National Coordinator: Dr. M. Stanojevic

26. National Reference Center for HIV/AIDS prevention, Slovak Medical University, Bratislava, Slovakia **
National Coordinator: Dr. D. Stanekova

27. Slovenian HIV/AIDS Reference Center, University of Ljubljana, Ljubljana, Slovenia **
National Coordinator: Prof. Dr. M. Poljak

28. Institut de Recerca de la SIDA – *irsiCaixa*, Hospital Universitari Germans Trias i Pujol Universitat Autònoma de Barcelona, Badalona, Spain **
National Coordinator: Dr. Bonaventura Clotet

29. Dept of Microbiology, Tumor and Cell Biology, Karolinska Institutet and Dept of Virology, Swedish Institute for Infectious Disease Control, Solna, Sweden **
National Coordinator: Prof. Dr. Jan Albert

30. Les Hopitaux Universitaires de Geneve, Department of Internal Medicine, Division of Infectious Diseases, Geneva, Switzerland
National Coordinator: Dr. S. Yerly

31. Ukrainian AIDS Center of Ministry of Health,Department of National laboratory Ukrainian AIDS Center, Kiev, Ukraine
National Coordinator: Dr. Olga Kravchenko

32. Department of Windeyer Institute, Royal Free Hospital and University College Medical School, London, United Kingdom
National Coordinator: Dr. Deenan Pillay
